# Capicua suppresses *YAP1* to limit tumorigenesis and maintain drug sensitivity in human cancer

**DOI:** 10.1016/j.celrep.2022.111443

**Published:** 2022-10-04

**Authors:** Ji Won Kim, Cuyler Luck, Wei Wu, Rovingaile Kriska Ponce, Yone Kawe Lin, Nehal Gupta, Ross A. Okimoto

**Affiliations:** 1 Department of Medicine, University of California, San Francisco, San Francisco, CA, USA; 2 Jeju Research Institute of Pharmaceutical Sciences, College of Pharmacy, Jeju National University, Jeju, Republic of Korea; 3 Helen Diller Family Comprehensive Cancer Center, University of California, San Francisco, San Francisco, CA, USA; 4 Lead contact

## Abstract

Inactivation of Capicua (CIC) or upregulation of yes-associated protein 1, YAP1, leads to broad RAS-RAF-MEK-ERK inhibitor resistance and tumor progression in multiple human cancers. Despite these shared malignant phenotypes, it remains unclear whether CIC and YAP1 are mechanistically linked. Here, we show that the ERK-regulated transcription factor CIC can directly repress *YAP1* expression through non-consensus GGAAGGAA DNA-binding motifs in a proximal *YAP1* regulatory element. Through binding at GGAA repeats, CIC regulates *YAP1* transcriptional output in both normal and human cancer cells. Silencing *YAP1* in *CIC*-deficient cells restores MAPK inhibitor sensitivity and suppresses tumor growth. Thus, we uncover a molecular link between the MAPK-ERK effector CIC and *YAP1* in human cells and established YAP inhibition as a strategy to target CIC-deficient cancers.

## In brief

Kim et al. show that the transcriptional repressor Capicua (CIC) regulates *YAP1* expression in human cancer. CIC-mediated *YAP1* suppression occurs through non-consensus GGAA repeat motifs. CIC loss increases *YAP1* expression to drive tumor progression. YAP inhibition in CIC-deficient cancers overcomes drug resistance and limits tumor growth.

## Graphical Abstract

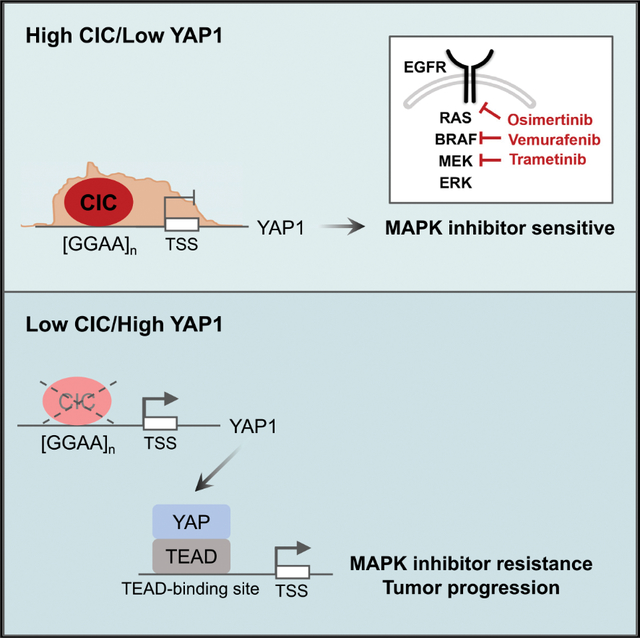

## INTRODUCTION

The mitogen-activated protein kinase (MAPK) pathway is a signaling cascade involved in cellular growth and differentiation (Barbosa et al., 2021). MAPK pathway activation occurs through binding of specific growth factors to cognate receptor kinases that recruit RAS to activate downstream substrates, including RAF, MEK, and ERK (Barbosa et al., 2021). Activated ERK propagates growth-dependent signals into functional phenotypes through regulation of cytoplasmic and nuclear substrates, including transcription factors (TFs) (Hollenhorst, 2012; Lavoie et al., 2020). In cancer, genetic and non-genetic alterations in key MAPK substrates hyperactivate this cascade, leading to tumor growth and survival (Barbosa et al., 2021; Hollenhorst, 2012; Lavoie et al., 2020). Recent precision-based therapies limit flux through RAS-RAF-MEK-ERK, improving outcomes for cancer patients (Moscow et al., 2018). Interestingly, mechanisms that lead to MAPK inhibitor resistance commonly converge and reactivate the terminal substrate, ERK, which can promote tumor growth and drug resistance through direct regulation of target TFs. One example is the transcriptional repressor Capicua (CIC), which silences ERK transcriptional targets (Futran et al., 2015; Jimenez et al., 2012; Keenan et al., 2020; Kim et al., 2021; Okimoto et al., 2017). Loss of CIC in development and cancer can partially reactivate and phenocopy RAS-RAF-MEK-ERK activation (Ajuria et al., 2011; Liao et al., 2017; Roch et al., 2002; Tseng et al., 2007). Moreover, activated ERK can functionally suppress CIC through phosphorylation, leading to CIC degradation and de-repression of target genes (Grimm et al., 2012; Keenan et al., 2020; Okimoto et al., 2017; Paul et al., 2020; Weissmann et al., 2018). Interestingly, inactivation of CIC has also been linked to RAS-RAF-MEK inhibitor resistance (Kim et al., 2021; Liao et al., 2017; Wang et al., 2017). The mechanisms of how CIC loss confers resistance to MAPK inhibition are not well-defined. Our studies identify the Hippo effector, *YAP1*, as a target of CIC potentially through non-consensus GGAA repeat sites and demonstrate that CIC and YAP cooperate to control drug resistance and tumor progression in human cancer.

## RESULTS

### Capicua controls YAP1 expression in human cancer cells

**Figure 1.**
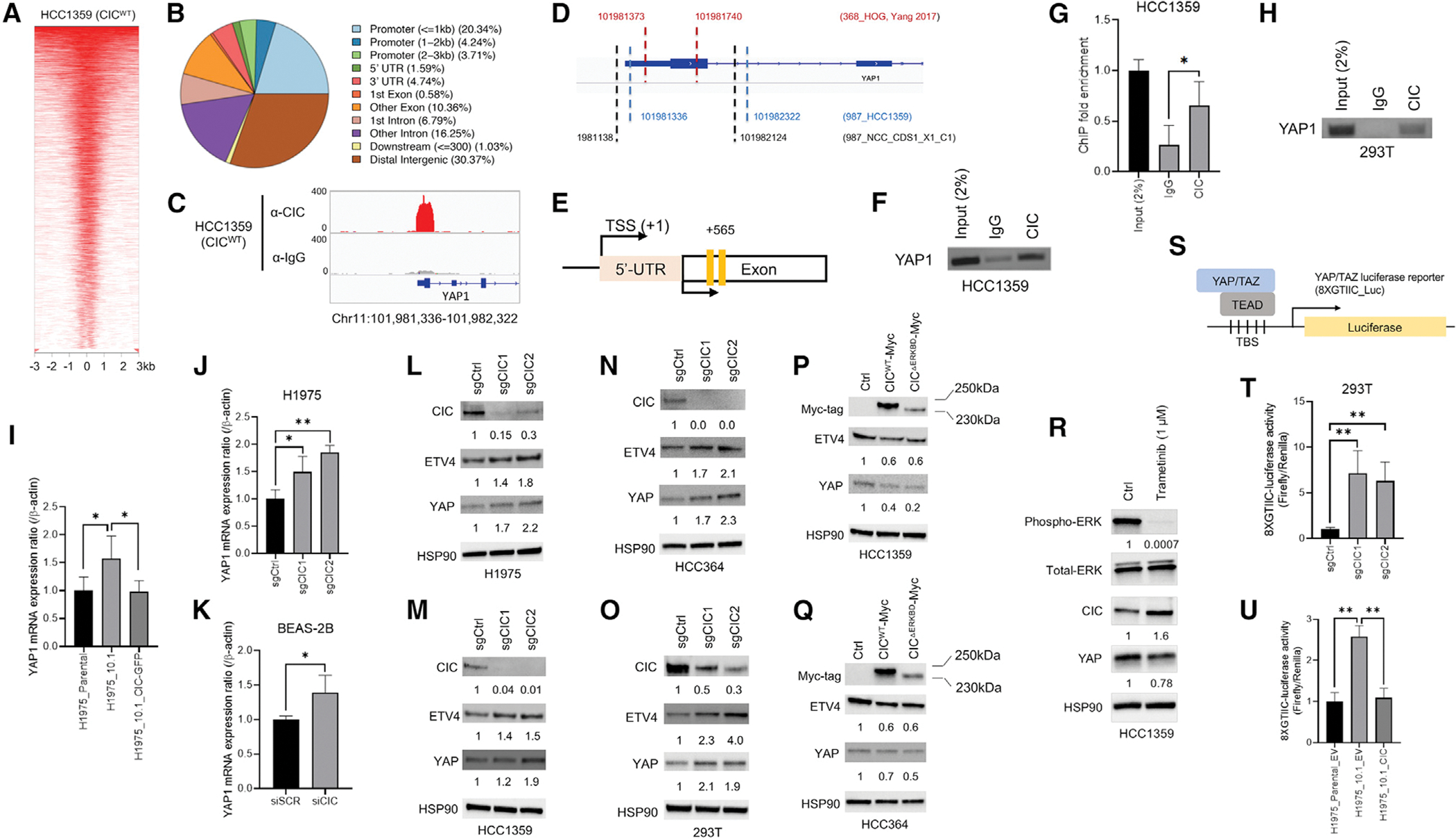
Capicua represses *YAP1* (A) Heatmap of CIC peaks relative to transcription start sites (upstream −3 kb to downstream +3 kb) in HCC1359s. (B) % of CIC peaks located at genomic regions. (C) ChIP-seq track from HCC1359s demonstrating CIC occupancy (upper red) of a *YAP1* RE. Immunoglobulin (Ig)G control (lower gray). (D) Alignment of WT-CIC (HCC1359 and HOG) and CIC-DUX4 (NCC_CDS1_X1_C1) ChIP peaks. Genomic coordinates of peaks indicated with dashed lines. (E) *YAP1* RE with locations of ChIP-PCR primers (yellow bars). (F–H) ChIP-PCR showing CIC occupancy on *YAP1* in HCC1359 (F and G) (n = 3) and HEK293T (H) cells. *p < 0.05. (I) Relative *YAP1* expression in H1975, H1975_10.1, and H1975_10.1 expressing GFP-CIC. *p < 0.05. (J) Relative *YAP1* expression in H1975s expressing sgCtrl, sgCIC1, or sgCIC2. *p < 0.05, **p < 0.01. (K) Relative *YAP1* in BEAS-2Bs expressing scramble (siSCR) or siCIC. *p < 0.05. (L–Q) Immunoblots (IB) of CIC, ETV4, and YAP in H1975 (L), HCC1359 (M), HCC364 (N), and HEK293T (O) expressing sgCtrl, sgCIC1, or sgCIC2. IB of CIC, ETV4, and YAP in HCC1359 (P) and HCC364 (Q) cells expressing control, CIC^WT^, or CIC^ΔERKBD^. (R) IB of pERK, tERK, CIC, and YAP in HCC1359s treated with trametinib (1 μM, 24 h). (S) Schematic of YAP/TAZ reporter. (T) Relative YAP/TAZ reporter activity in HEK293Ts expressing sgCtrl, sgCIC1, or sgCIC2. **p < 0.01. (U) Relative YAP/TAZ reporter activity in H1975 + EV, H1975_10.1 + EV, and H1975_10.1 + CIC expression. **p < 0.01. Error bars represent SD.

To uncover the mechanisms of drug resistance in CIC-deficient cancers, we performed chromatin immunoprecipitation followed by sequencing (ChIP-seq) in human lung cancer (LC) cells (HCC1359). This revealed global CIC-binding sites with 79,359 peaks (p < 0.05) at diverse genomic regions (Figures 1A and 1B). This analysis identified a significant peak localized to the proximal regulatory element (RE) of *YAP1* (Figure 1C). Aligning our findings with a publicly available ChIP-seq dataset (Yang et al., 2017) in human oligodendroglioma (HOG) cells, we observed a similar CIC peak that overlapped with the genomic coordinates in HCC1359s (Figure 1D). Moreover, ChIP-seq analysis of the Capicua-containing fusion, CIC-DUX4, in patient-derived NCC_CDS1_X1_C1 cells again revealed an overlapping peak within the proximal *YAP1* RE (Figures 1D, S1A, and S1B). CIC-DUX4 retains CIC target gene specificity, but through neomorphic function gains activating capacity (Kawamura-Saito et al., 2006; Lin et al., 2020; Okimoto et al., 2019; Yoshimoto et al., 2017). Notably, CIC-DUX4 expression regulated YAP expression (Figures S1C and S1D). These findings suggest that wild-type (WT) CIC potentially binds to a *YAP1* proximal RE to suppress YAP-mediated transcription. Since YAP upregulation confers a survival dependence in RAS-RAF-MEK inhibitor resistant human cancers (Kapoor et al., 2014; Lin et al., 2015; Shao et al., 2014; Su et al., 2021; Zhang et al., 2014), we hypothesized that WT-CIC represses *YAP1* to maintain a drug sensitive state. To explore this, we first confirmed that WT-CIC binds to the *YAP1* RE by ChIP-PCR in human LC cells and HEK293T cells (Figures 1E–1H and S1E–S1H). Next, we modulated *YAP1* levels through CIC expression using EGFR mutant H1975_10.1 cells, which are EGFR inhibitor resistant derivatives of the parental H1975 LC cell line (Okimoto et al., 2017; Walter et al., 2013). H1975_10.1 cells are deficient for *CIC* through homozygous deletion (Okimoto et al., 2017), while H1975s are CIC sufficient. We genetically reconstituted *CIC* into H1975_10.1 cells and noted that *YAP1* (and known CIC targets *ETV1* and *ETV4*) expression decreased compared with H1975_10.1 cells expressing an empty vector (EV) control to levels similar to H1975s (Figures 1I and S1I–S1K). Using H1975s, we then engineered two independent *CIC* knockout (KO) lines that increased *YAP* expression compared with controls (Figures 1J and S1L–S1M). To mitigate off-target effects of our guide RNAs, we silenced *CIC* through RNAi in bronchial epithelial cells (BEAS-2B), HCC364^BRAF(V600E)^, A549^KRAS(G12S)^, and SW1573^KRAS(G12C)^ mutant LC cells and observed increased *YAP1* expression in *CIC* knockdown (KD) cells compared with control (Figures 1K and S1N–S1T). To demonstrate that CIC is sufficient to suppress *YAP1* expression, we rescued a loss-of-function *CIC*^F780S^ variant in AGS gastric cancer cells and observed a decrease in *YAP1* expression (Okimoto et al., 2017) (Figures S1U–S1W). We further observed an increase in YAP protein levels in H1975, HCC1359, HCC364, and HEK293T cells expressing two independent *CIC* guide RNAs (sgCIC1 and sgCIC2) compared with sgCtrl (Figures 1L–1O). In contrast, YAP expression decreased upon overexpression (OE) of WT-CIC and was further suppressed with expression of a CIC variant that abrogates the ERK-binding domain (resistant to ERK-mediated degradation) (Futran et al., 2015), CIC^ΔERKBD^, in HCC1359 and HCC364 cells (Figures 1P, 1Q, S1X, and S1Y). Since increased MEK-ERK activity decreases CIC protein expression, we used trametinib (MEK inhibitor) to block MEK-ERK flux in HCC1359 cells. We observed an increase in CIC expression, which corresponded to a decrease in YAP expression upon trametinib treatment (Figure 1R). In order to test if CIC could regulate YAP targets, we used a Luc-based YAP/TEAD reporter assay (8XGTIIC) (Dupont et al., 2011) (Figure 1S) and observed increased activity upon *CIC* KO in HEK293T cells (Figure 1T). Moreover, CIC rescue decreased YAP/TEAD reporter activity in CIC-deficient H1975_10.1 LC cells compared with EV controls (Figure 1U). Next, we analyzed mRNA levels of *CIC*, *ETV4*, and *YAP1* in colorectal cancer (HT-29) and melanoma (A375) cells, expressing sgCtrl, sgCIC1, or sgCIC2. *YAP1* increased in HT-29 and A375 cells that expressed sgCIC1 or sgCIC2 compared with sgCtrl (Figures S2A–S2F). In addition, mRNA expression of known YAP targets (*CTGF*, *CYR61*, *CCND1*, and *BIRC5*) increased in HCC1359s expressing sgCIC1 and sgCIC2, compared with sgCtrl control (Figures S2G–S2J).

To determine how CIC regulates YAP in the context of the core Hippo kinases, LATS1 and LATS2, we engineered LATS1/2 deficient HCC1359s and tested the effects of CIC on YAP expression. Specifically, we silenced *LATS1* and *LATS2* in HCC1359 cells using CRISPR-Cas9 (sgLATS2) and RNAi (siLATS1) ± CIC expression. YAP expression did not significantly change in LATS1- and/or LATS2-deficient HCC1359s (Figure S2K). Interestingly, we observed that *CIC* KO increased YAP levels irrespective of LATS1 and/or LATS2 expression (Figure S2L). These findings suggest that the effects of CIC on YAP are in part, independent, or work in parallel to the known YAP regulators, LATS1, and/or LATS2 in HCC1359 cells.

### CIC binds and regulates target gene expression, including *YAP1*, through GGAA repeats

**Figure 2.**
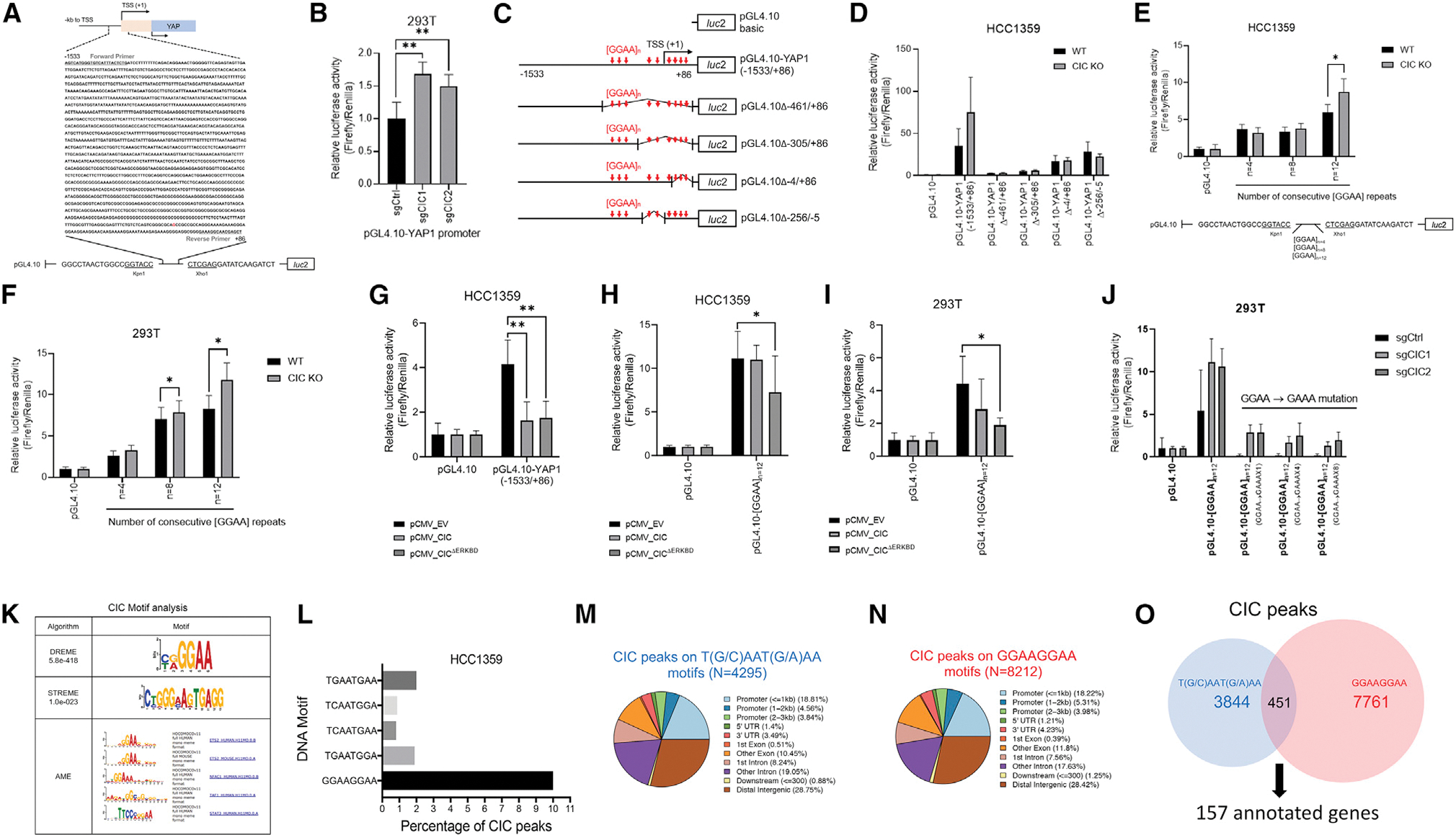
CIC binds and regulates YAP1 expression through non-consensus GGAAGGAA DNA motifs (A) pGL4.10 reporter construct containing the genomic sequence (−1,533 to +86) of *YAP1* RE occupied by CIC. (B) Relative pGL4.10-*YAP1* activity in HEK293Ts expressing sgCtrl, sgCIC1, or sgCIC2. **p < 0.01. (C) Control and mutant pGL4.10 with associated GGAA sites. (D) Relative pGL4.10-YAP1 activity in HCC1359s expressing either control or a mutant pGL4.10 Luc construct ± CIC (CIC^WT^ or CIC KO). (E) Relative Luc activity of pGL4.10 constructs containing GGAA repeats (n = 4, n = 8, n = 12) in HCC1359s comparing CIC^WT^ and CIC KO. *p < 0.05. (F) Relative Luc activity of pGL4.10 constructs containing GGAA repeats (n = 4, n = 8, n = 12) in HEK293Ts comparing CIC^WT^ and CIC KO. *p < 0.05. (G) Relative Luc activity of the pGL4.10-YAP1 (−1,533/+86) and pGL4.10 control in HCC1359s expressing CIC^WT^ or CIC^ΔERKBD^. *p < 0.05, **p < 0.01. (H and I) Relative Luc activity of the pGL4.10-[GGAA]_n=12_ and pGL4.10 control in HCC1359 cells (H) or HEK293Ts (I) overexpressing CIC^WT^ or CIC^ΔERKBD^ compared with EV. *p < 0.05, **p < 0.01. (J) Relative Luc activity of the pGL4.10-[GGAA]_n=12_ constructs containing GGAA→GAAA mutation repeats (n = 1, n = 4, n = 8) in HEK293Ts expressing sgCtrl, sgCIC1, or sgCIC2. (K) DREME, STREME, and AME analysis in HCC1359s identify GGAA sequences as putative CIC-binding sites. (L) % of total CIC peaks in HCC1359s that align to [GGAA]_n_ (n ≥ 2, designated as GGAAx2+ thereafter) and T(G/C)AAT(G/A)AA DNA motifs. (M and N) % of T(G/C)AAT(G/A)AA (M) or GGAAx2+ (N) associated CIC peaks at genomic regions in HCC1359s. (O) Venn diagram comparing CIC peaks at T(G/C)AAT(G/A)AA or GGAAx2+ DNA motifs; 451 shared peaks mapping to 157 genes. Error bars represent SD.

The CIC-binding region within the *YAP1* RE did not contain the canonical CIC DNA-binding motif, T(G/C)AAT(G/A)AA (Jimenez et al., 2012). This prompted us to map the region required for CIC-regulated transcriptional activity. We first generated a luciferase (Luc)-based *YAP1* reporter construct (pGL4.10) that contained the putative CIC-binding region (Figure 2A). We then validated that CIC loss increases *YAP1* reporter activity in HEK293T cells (Figure 2B). Next, we deleted regions within the pGL4.10-*YAP1* reporter construct and noted that the 3′-end was required for full activity in HCC1359 cells (Figures 2C and 2D). Further analysis revealed an increased frequency of GGAA repeats [GGAA]_n_. To test whether CIC loss could enhance reporter activity through repeat GGAA sequences, we engineered Luc-based constructs that contained increasing numbers of GGAA repeats and observed increased activity in HCC1359 and HEK293T cells upon *CIC* KO compared with control (Figures 2E and 2F). In contrast, OE of CIC^WT^ or CIC^ΔERKBD^ decreased Luc activity from a pGL4.10 construct containing *YAP1* or 12-GGAA repeats ([GGAA]_n=12_) in HCC1359 and HEK293T cells (Figures 2G–2I). We next performed guanine→alanine mutagenesis to perturb either 1, 4, or 8 GGAA sites in the pGL4.10-[GGAA]_n=12_ construct. Disruption of the GGAA repeats decreased *YAP1* reporter activity (Figure 2J). To gain a global assessment of CIC binding at GGAA repeat sites, we performed motif-based sequence analysis using three motif enrichment algorithms (DREME, STREME, and AME), which revealed both *de novo* and known motifs containing GGAA sequences as highly significant in our HCC1359 ChIP-seq dataset (Figure 2K). To understand how CIC peaks associate with GGAA repeats compared with the consensus T(G/C)AAT(G/A)AA DNA-binding motif, we performed a comparative ChIP-seq-based analysis in HCC1359 cells. This analysis identified CIC peaks (q < 0.05) at 8,212 genomic sites with [GGAA]_n_ (n ≥ 2) (denoted as GGAAx2+ thereafter) that corresponded to 2,053 annotated genes, accounting for 10% of all CIC peaks in our dataset (Figure 2L). By comparison, we observed 4,295 genomic loci (corresponding to 1,185 annotated genes) that mapped to the T(G/C)AAT(G/A)AA CIC DNA-binding octamer, accounting for 5.63% of all CIC peaks (Figure 2L and Table S1). The fraction of CIC peaks binding to either GGAAx2+ or T(G/C)AAT(G/A)AA sites at defined genomic regions were comparable (Figures 2M–2O). Interestingly, there were 451 genomic sites (157 annotated genes) that contained both GGAAx2+ and T(G/C)AAT(G/A)AA CIC motifs (Figure 2O and Table S2). Comparative Pathway Analysis (Kyoto Encyclopedia of Genes and Genomes) using the 1,185 and 2,053 genes mapped to T(G/C)AAT(G/A)AA or GGAAx2+ sites, respectively, converged on MAPK signaling in addition to other diverse cellular functions (Figures S3A and S3B). In contrast, genes shared (n = 157) between T(G/C)AAT(G/A)AA and GGAAx2+ sites associated with neurotransmitters/synapse (Figure S3C). These findings indicate that CIC can bind and regulate genes, including *YAP1*, through GGAA repeat sequences to modulate diverse cellular functions. To explore the potential overlap between CIC binding and gene expression, we integrated our ChIP-seq analysis with RNA sequencing (RNA-seq) data from HCC1359 cells ± CIC expression. As noted previously, a significant fraction of CIC peaks were not associated with annotated genes. This suggests that CIC binds to distal intergenic regions across the genome. Using the ChIP-guided annotated gene set that contained either the conventional CIC-binding octamer, GGAA repeats, or both CIC octamer and GGAA repeats, we identified gene expression changes (Log2FC > 1.5 and FDR<0.2) that overlapped with these CIC peaks (Figure S3D).

### CIC and YAP cooperate to control drug resistance and tumor progression in human cancer

**Figure 3.**
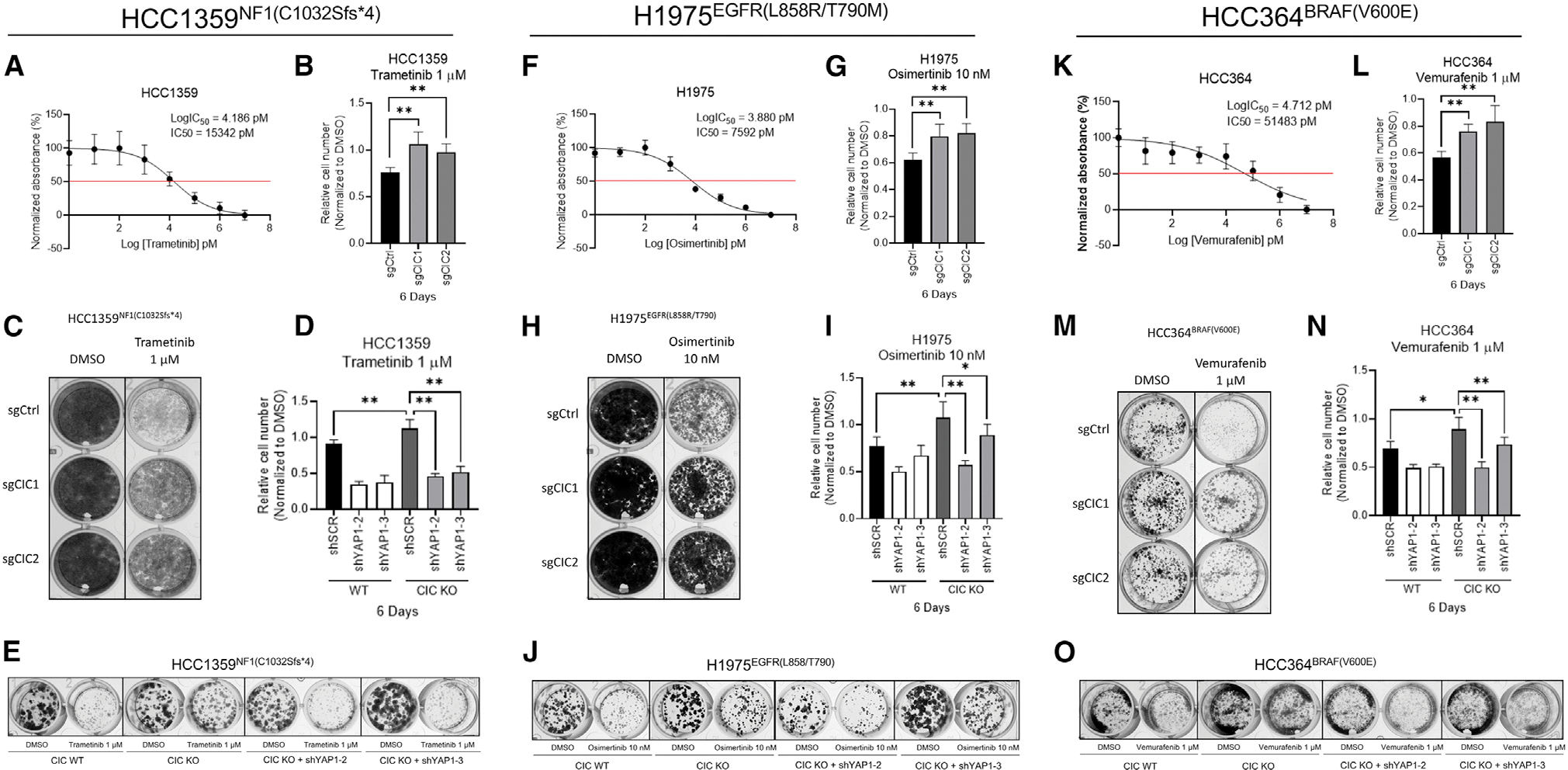
YAP in CIC-deficient human lung cancer drives tumor progression and resistance to MAPK inhibitors (A, F, and K) CTG viability curves in HCC1359 (A), H1975 (F), and HCC364 (K) following treatment with trametinib (tram), Osimertinib (osi), or vemurafenib (vem), respectively. (B, G, and L) Relative cell number of HCC1359 (B), H1975 (G), and HCC364 (L) cells expressing sgCtrl, sgCIC1, or sgCIC2 and treated (6 d) with tram, osi, or vem, respectively. **p < 0.01. (C, H, and M) Crystal violet (CV) assay of HCC1359 (C), H1975 (H), and HCC364 (M) cells expressing sgCtrl, sgCIC1, or sgCIC2 treated (10 d) with tram, osi, or vem, respectively. (D, I, and N) Relative cell number of HCC1359 (D), H1975 (I), and HCC364 (N) cells expressing sgCtrl, sgCIC1, or sgCIC2 ± *YAP1* KD (shYAP1–2 and shYAP1–3) treated (6 d) with tram, osi, or vem, respectively. *p < 0.05, **p < 0.01. (E, J, and O) CV assay of HCC1359 (E), H1975 (J), and HCC364 (O) cells expressing sgCtrl, sgCIC1, or sgCIC2 ± *YAP1* KD (shYAP1–2 and shYAP1–3) treated (10 d) with tram, osi, or vem, respectively. Error bars represent SD in all figures.

Since both CIC loss and increased YAP expression contribute to RAF-MEK-ERK inhibitor resistance in human LC, we hypothesized that increased YAP could be regulating drug resistance in CIC-deficient cancers. To test this, we treated HCC1359^NF1(C1032Sfs^*^4)^, H1975^EGFR(L858R/T790M)^, and HCC364^BRAF(V600E)^ LC cells with cognate targeting agents (HCC1359-trametinib, H1975-osimertinib, HCC364-vemurafenib) (Figures 3A, 3F, and 3K). Consistent with prior studies (Liao et al., 2017; Okimoto et al., 2017; Wang et al., 2017), *CIC* KO conferred inhibitor resistance in these LC subsets (Figures 3B, 3C, 3G, 3H, 3L, and 3M). The decreased drug sensitivity observed upon *CIC* KO was rescued with *YAP1* KD (Lin et al., 2015) in all molecular subsets of LC as measured by CellTiter-Glo (CTG) and crystal violet (CV) assays (Figures 3D, 3E, 3I, 3J, 3N, and 3O). To test the potential clinical impact of YAP inhibition in CIC-deficient LC we used the YAP inhibitor, verteporfin (Feng et al., 2016) in HCC1359 cells ± CIC expression. Verteporfin treatment decreased YAP expression (Figure S4A) and enhanced the trametinib-mediated effect on CIC-deficient (*CIC* KO) HCC1359 cells compared with control (Figures S4B–S4D).

### YAP inhibition is a therapeutic strategy in CIC-deficient human cancers

**Figure 4.**
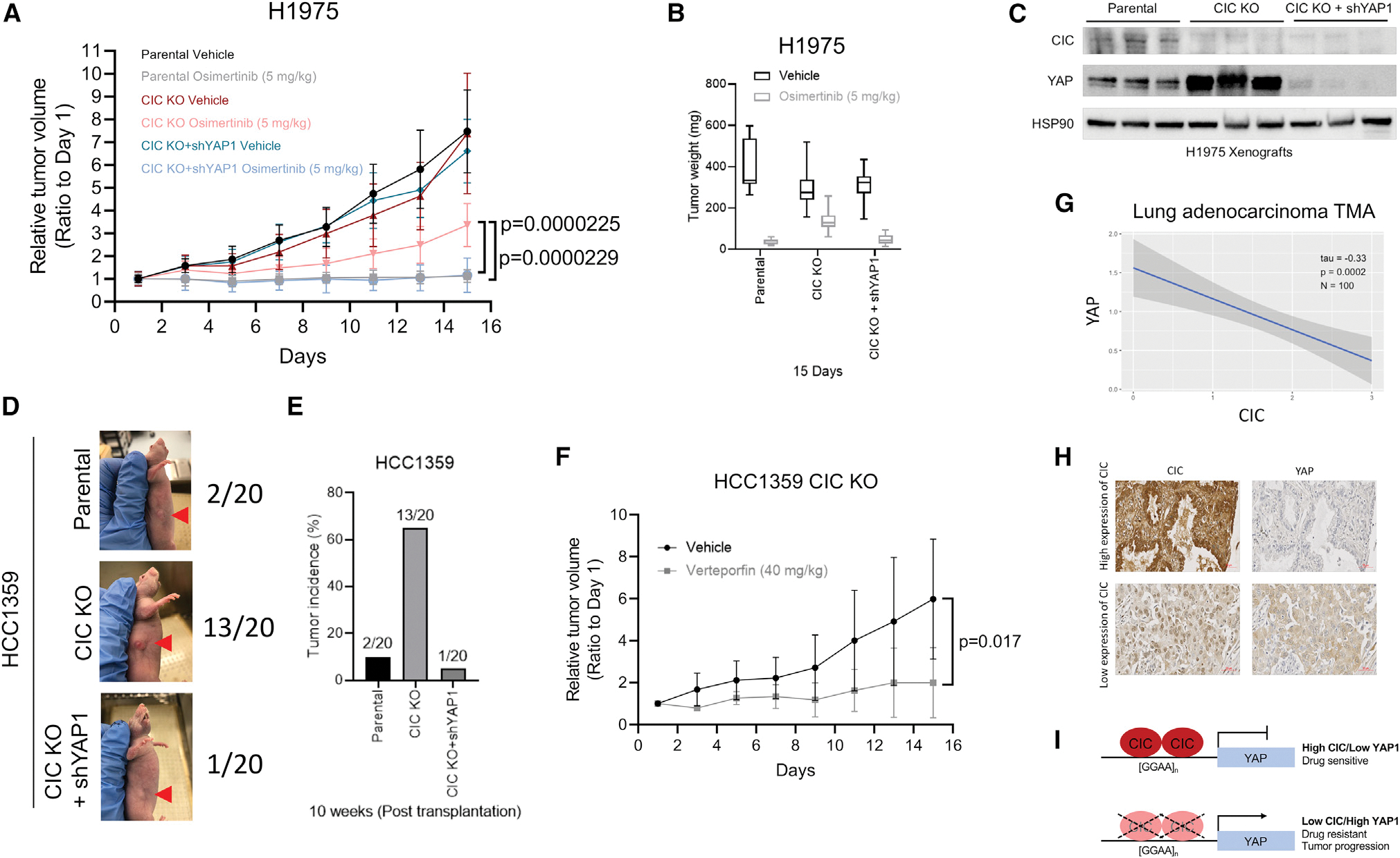
MAPK inhibitor resistance and tumor growth in CIC-deficient lung cancer are overcome through YAP1 inhibition *in vivo* (A) H1975 xenografts comparing vehicle or osimertinib-treated H1975, H1975 with *CIC* KO, or H1975 with *CIC* KO + *YAP1* KD (n = 10). (B) Tumor weights from mice harboring H1975, H1975 *CIC* KO, or H1975 *CIC* KO + *YAP1* KD ± osimertinib. (C) Immunoblots of CIC, YAP, and HSP90 expression in H1975 xenografts derived from tumors of different groups in (A). (D) Representative figures of parental, *CIC* KO, or *CIC* KO + *YAP1* KD in HCC1359 tumor-bearing mice. (E) Bar graph comparing the incidence of HCC1359 (n = 2/20), HCC1359 *CIC* KO (n = 13/20), or HCC1359 *CIC* KO + *YAP1* KD (n = 1/20) tumor formation in mice. (F) Relative growth of HCC1359 *CIC* KO tumors treated with vehicle or verteporfin. (G) Correlation plot between CIC and YAP expression from LC specimens (tau = −0.33, p = 0.0002, n = 100). (H) Representative images of CIC and YAP-stained LC tissue. Scale bar, 50 μm. (I) Model of CIC-mediated repression of *YAP1* and phenotypes. Error bars represent SD.

We next wanted to determine the efficacy of targeting *YAP1* to restore osimertinib sensitivity in CIC-deficient LC. Accordingly, we first generated subcutaneous (SC) xenografts in immunodeficient mice that harbored H1975 cells or H1975 cells with *CIC* KO ± *YAP1* KD. Consistent with our *in vitro* findings, we observed decreased osimertinib sensitivity in mice bearing CIC-deficient H1975s. Osimertinib sensitivity was restored upon *YAP1* KD in H1975 *CIC* KO cells (Figures 4A–4C and S4E). We next assessed the establishment of HCC1359 SC xenografts *in vivo* and observed infrequent (10%, 2 of 20) tumors in mice harboring HCC1359s. CIC loss increased the incidence of tumors in mice bearing HCC1359 *CIC* KO xenografts (Figures 4D and 4E). The increased tumor formation rate in CIC-deficient HCC1359 mice was attenuated with *YAP1* KD (Figures 4D and 4E). Following treatment of mice harboring CIC-deficient (*CIC* KO) HCC1359 tumors with verteporfin, we observed a decrease in tumor growth compared with control (Figure 4F). Collectively, our findings suggest that increased YAP expression can potentially mediate drug resistance and tumor growth in CIC-deficient LCs. To augment these findings, we assessed if CIC expression is anti-correlated with YAP protein expression in human LC specimens. Specifically, we performed immunohistochemical staining (IHC) in a human LC tissue microarray (TMA). We observed an anti-correlation between CIC and YAP protein expression (tau = −0.33, p = 0.0002, n = 100), with decreased YAP expression in CIC-High specimens (IHC score 2 or 3, n = 50) compared with CIC-Low specimens (IHC score 0 or 1, n = 50) (Figures 4G and 4H). Collectively, these data corroborate our *in vitro* findings and support targeting YAP in CIC-deficient cancers to overcome drug resistance and to limit tumor progression.

## DISCUSSION

We have uncovered a CIC-dependent molecular link between MAPK-ERK signaling and *YAP1* transcriptional programs. Through binding and functional regulation at GGAA repeats, CIC suppresses *YAP1* expression. Inactivation of CIC increases *YAP1* expression, which confers tumor growth, survival, and resistance to MAPK inhibitors.

We identified GGAA repeats within the REs of putative target genes as potential global sites for CIC binding. These observations could have a broad impact in understanding how CIC regulates divergent (and convergent) transcriptional programs across human development and cancer. Importantly, we did not find evidence of CIC binding to the *YAP1* RE in mouse cells (Weissmann et al., 2018; Yang et al., 2017). Thus, CIC binding to GGAA repeats may lack evolutionary conservation and be species-specific. In addition, it remains unclear how CIC loss leads to transcriptional activation of target genes including *YAP1*. One potential explanation is that WT-CIC occupies and thus competes with positive ETS factors for GGAA sites—a consensus ETS-binding motif (Karim et al., 1990; Wei et al., 2010). Others have identified “low-affinity” AT sites that associate with CIC binding in *Drosophila* (Papagianni et al., 2018). These studies suggest that cell intrinsic factors such as signaling pathways can play a role in defining how and when CIC can bind to and regulate target genes. Our findings align with these studies and suggest that the interaction between CIC and GGAA repeats may not only be cell context specific, but also low-affinity species-specific binding sites.

### Limitations of the study

Our *in vivo* models revealed phenotypic differences between our cell lines. Specifically, H1975 and HCC1359 do not align in their ability to induce tumor formation in immunodeficient mice, a system influenced by multiple factors that may or may not work to suppress tumor initiation of HCC1359 relative to H1975 cells. These factors include a partially intact immune system and a microenvironment that is both unique to the host and absent in *in vitro* models. These tumor cell to microenvironmental factors are further compounded by intrinsic genetic, non-genetic, and cellular differences between HCC1359 and H1975 cells that extend beyond the scope of our work.

## STAR★METHODS

### RESOURCE AVAILABILITY

#### Lead contact

Requests for resources and reagents should be directed to and will be fulfilled by the lead contact, Ross Okimoto (ross.okimoto@ucsf.edu).

#### Materials availability

All materials are available from the lead contact upon request.

#### Data and code availability

This paper analyzes existing, publicly available data as referenced in the manuscript. All additional data are available from the lead contact upon request.

This paper does not report original code.

Any additional information required to reanalyze the data reported in this paper is available from the lead contact upon request.

### EXPERIMENTAL MODEL AND SUBJECT DETAILS

#### Animal models: Subcutaneous tumor xenograft assays

Four-weeks old female nude (NU/J) mice were purchased from Jackson Laboratory and were six-weeks old at time of experiment. For subcutaneous xenotransplantation, H1975 (1×10^6^ cells/flank) and HCC1359 (5×10^6^ cells/flank) cells were re-suspended in a mixture of 50% PBS/50% Matrigel matrix and injected into the right and left flanks of mice. When the average tumor volume of H1975 reached 75 mm3, the mice were randomized into 2 groups to receive the following treatments: (a) Vehicle (2% DMSO, 20% PEG300, 2% Tween-80, 76% PBS); (b) Osimertinib (5 mg/kg, daily, p.o). The CIC KO HCC1359 tumor-bearing mice were also randomized into 2 groups: (a) Vehicle (2% DMSO, 20% PEG300, 2% Tween-80, 76% PBS); (b) Verteporfin (40 mg/kg, every 2 days, i.p). Tumor volume and body weight were measured every 2 days. Tumor volume was determined using caliper measurements of tumor length (*L*) and width (*W*) according to the formula *V* = (*L* × *W*^2^) × 0.52. Mice were observed post-procedure for 1–2 hours, and body weight and wound healing were monitored per IACUC protocol.

#### Animal study approval

For tumor xenograft studies, specific pathogen-free conditions and facilities were approved by the American Association for Accreditation of Laboratory Animal Care. Surgical procedures were reviewed and approved by the UCSF Institutional Animal Care and Use Committee (IACUC), protocol #AN178670–03.

#### Cell lines

Cell lines were cultured as recommended by the American Type Culture Collection (ATCC). H1975, HEK293T, HCC1359, HCC364, A549, SW1573 cells were purchased from ATCC. H1975_10.1 (CIC null) cells were derived from parental H1975 cells as previously described (Walter et al., 2013). NCC_CDS1_X1_C1 cells were generated as patient-derived cell lines and validated previously (Oyama et al., 2017). HCC1359, HCC364, A549, SW1573 and NCC_CDS1_X1_C1 cells were grown RPMI 1640 media supplemented with 10% FBS, 100 IU/mL penicillin and 100 μg/mL streptomycin, and H1975, H1975_10.1, A375 and HEK293T cells were grown DMEM media supplemented with 10% FBS, 100 IU/mL penicillin and 100 μg/mL streptomycin, respectively. HT-29 cells were grown McCoy’s 5a media supplemented with 10% FBS, 100 IU/mL penicillin and 100 μg/mL streptomycin. All cell lines were maintained at 37°C in a humidified atmosphere at 5% CO2.

### METHOD DETAILS

#### Stable CRISPR/Cas9 knockout of CIC

Two sgRNAs targeting *CIC* were previously validated and were gifts from William Hahn Addgene (#74959 and #74953). The CRISPR/Cas9 lentivirus was produced with a plasmid containing Cas9 and either of the two sgRNAs directed at CIC (9 μg), pMD2.G VSV-G envelope expressing plasmid (1 μg), pCMV-dR8.91 packaging vector (8 μg) and FuGENE 6 (Cat# E2692, Promega) in HEK293T cells. Target cells were infected with the sgRNAs containing lentivirus for 48 h and selected with 10 μg/mL Blasticidin (Cat# A1113903, Thermo Fisher Scientific) for 1 week. The surviving cells were continuously cultured for subsequent experiments.

The CRISPR/Cas9 lentivirus using the LV05 vector (U6-gRNA:EF1a-Cas9+FLAG-2A-Puro) containing the sgRNA targeting LATS2 (human LATS2, 5′-TACGCTGGCACCGTAGCCCT-3′) was produced in HEK293T cells as well.

#### Gene knockdown and overexpression assays

ON-TARGET plus Scramble, CIC siRNA (#L-015185-01-0005), and LATS1 siRNA (#L-004632-00-0005) were obtained from GE Dharmacon and transfection was performed with RNAiMax transfection reagent (Thermo Fisher Scientific).

Two shRNAs targeting *YAP1* were previously validated and a kind gift from T. Bivona (Lin et al., Nature Genetics, 2015). shScramble was purchased from Sigma Aldrich.

Overexpression. pCMV-CIC with myc-tag was purchased from Origene and validated previously (Okimoto et al., 2017).

Lentiviral plasmid containing the CIC-GFP insert was purchased from Origene and previously validated (Okimoto et al., 2017).

#### Drugs

Trametinib (Cat# HY-10999), osimertinib (Cat# HY-15772), vemurafenib (Cat# HY-12057), and verteporfin (Cat# HY-B0146) were purchased from Medchemexpress. Recombinant EGF was purchased from Peprotech.

#### Chromatin immunoprecipitation with sequencing (ChIP-Seq) and PCR

CIC immunoprecipitation was performed using HCC1359 and NCC_CDS1_X1_C1 cells. SimpleCHIP Enzymatic Chromatin IP Kit (Cat# 9003S, Cell Signaling Technology) was used with IgG (Cell signaling Technology) and CIC (Thermo Fisher Scientific) antibody per the manufacturer’s protocol. Paired-end 150bp (PE150) sequencing on a HiSeq platform was subsequently performed. ChIP-Seq peak calls were identified through Mode-based Analysis of ChIP-Seq (MACS).

For *YAP1* ChIP-PCR validation, primers were designed to flank a region (+565) in the proximal regulatory element of *YAP1*.

The primer sequences were as follows:

*YAP1* Forward Primer: 5’-GGACTCGGAGACCGACCTGGAG-3’

*YAP1* Reverse Primer: 5′-GCTCCGGCGGCTTGAAGAAGG-3’.

#### Mutagenesis

Q5 Site-Directed Mutagenesis Kit was purchased from New England BioLabs (#E0554) and performed according to the manufacturer’s protocol. The myc-tagged CIC plasmid was purchased from Origene and validated previously (Okimoto et al., 2017).

The CIC^ΔERKBD^ mutant was generated using the following primers:

Forward 5’-CTGGATTCAGCACCCGAGGACC-3’ and

Reverse 5′-CGCCTCCTTGCGCTCCGG-3’.

Annealing temperature set at 72 degrees Celsius. The sequence was verified by Sanger sequencing.

#### Luciferase promoter assay

pGL4.10-*YAP1* luciferase reporter assay. Cells were split into a 96-well plate to achieve 70% confluence on the day of transfection. For luciferase promoter assay, Dual-Luciferase reporter assay system (Car# E1910, Promega) was used per the manufacturer’s protocol. Briefly, a mixture containing FuGENE 6 transfection reagent, luciferase reporter plasmid DNA (FLuc 150 ng and RLuc 150 ng per well), and either control (empty vector), CIC^WT^ or CIC^ΔERKBD^ was added to each well. 48 h after transfection, cells were harvested and luciferase activity was measured. All transfections were performed in sextuplicate (X6).

A genomic sequence (−1,533 and +86) within the proximal *YAP1* regulatory element was cloned in the pGL4.10 luciferase vector using the Kpn1 and Xho1 restriction sites per the manufacturer’s protocol. Deletions were generated using the Q5 Site-Directed Mutagenesis Kit from New England BioLabs (#E0554).

The following primers were used to generate the pGL4.10-YAP1 (−1,533/+86) construct:

Forward: 5′-GATCGGTACCAGTCATGGGTGTC-3’.

Reverse: 5′-GATCCTCGAGAGCTCGTTGCCTTTC-3’.

The following primers were used to generate deletions in the pGL4.10 construct containing the genomic sequence extending from −1,533 to +86 (pGL4.10-*YAP1* (−1,533/+86):

pGL4.10-YAP1 (−461/+86).

Forward 5′-TATCAAGATCTGGCCTCG-3’.

Reverse 5′-GCCGAAAGAAGTGGAGAG-3’.

pGL4.10-YAP1 (−305/+86).

Forward 5′-TATCAAGATCTGGCCTCG-3’.

Reverse 5′-CAAACGATGGGTCCAATC-3’.

pGL4.10-YAP1 (−4/+86).

Forward 5′-TATCAAGATCTGGCCTCG-3’.

Reverse 5′-CCCGACTGAGACAGAAAC-3’.

pGL4.10-YAP1 (−256/−5).

Forward 5′-CGCAGCCGCCGCCAGGGAAAAG-3’.

Reverse 5′-CAGCCGGGCAGGGGCCCG-3’.

GGAA repeat promoter assays were generated by introducing [GGAA]_n=4_, [GGAA]_n=8_, [GGAA]_n=12_, [GGAA]_n=12(GGAA→GAAAX1)_, [GGAA]_n=12(GGAA→GAAAX4)_, [GGAA]_n=12(GGAA→GAAAX8)_ oligonucleotide sequences into the pGL4.10 plasmid using the Kpn1 and Xho1 restriction sites.

The YAP/TEAD (8XGTIIC) luciferase assay was a gift from Stefano Piccolo and purchased from Addgene (#34615).

#### Western blot analysis

All immunoblots represent at least two independent experiments. Adherent cells were washed and lysed with RIPA buffer supplemented with proteinase and phosphatase inhibitors. Proteins were separated by SDS-PAGE, transferred to Nitrocellulose membrane, and blotted with antibodies recognizing: CIC (Thermo Fisher Scientific), YAP, LATS1, LATS2, Myc-tag, total-ERK, phospho-ERK, HSP90, IgG (Cell Signaling), ETV4 (Santa Cruz).

Xenograft tumors harvesting. Subcutaneous xenografts were explanted on day 15 of treatment. Tumor explants were immediately immersed in liquid nitrogen and stored at −80 degrees. Tumors were disrupted with a mortar and pestle, followed by sonication in RIPA buffer supplemented with proteinase and phosphatase inhibitors. Proteins were separated as above. Antibodies to CIC (Thermo Fisher Scientific), YAP, and HSP90 (Cell Signaling) were used.

Quantification of immunoblots was performed using ImageJ software.

#### Real-time quantitative polymerase chain reaction (RT-Q-PCR)

Isolation and purification of RNA was performed using RNeasy Mini Kit (Qiagen). 500 ng of total RNA was used in a reverse transcriptase reaction with the SuperScript III first-strand synthesis system (Invitrogen). Quantitative PCR included three replicates per cDNA sample. Human CIC (Hs00943425_g1), YAP1 (Hs00902708_g1), ETV1 (Hs00951951_m1), ETV4 (Hs00383361_g1), CTGF (Hs00170014_m1), CYR61 (Hs00155479_m1), CCND1 (Hs00765553_m1), BIRC5 (Hs04194392_s1), and β-actin (Hs01060665_g1) were amplified with Taqman gene expression assays (Applied Biosystems). Expression data were acquired using an ABI Prism 7900HT Sequence Detection System (Applied Biosystems). Expression of each target was calculated using the 2-ΔΔCt method and expressed as a relative mRNA expression.

#### ChIP-seq analysis

The reads from ChIP-Seq were mapped to reference genome hg19, Model-based Analysis of ChIP-Seq (MACS2) algorithm (version 2.2.1) [PMID:18798982] was used for the peak calling. The significant peaks between protein of interest (CIC) and background IgG bindings were identified based on adjusted p value less than 0.005. The peaks were further annotated including functional enrichment analysis of target genes using R package ChIPseeker [PMID:25765347] algorithm.

For global comparative CIC peak binding we systematically searched for CIC peak sequences that contained the GGAAGGAA, TGAATGAA, TCAATGGA, TCAATGAA, or TGAATGGA motifs with in-house bash script.

Motif Analysis was performed using the DREME (Bailey, 2011), STREME (Bailey, 2021), and AME (McLeay and Bailey, 2010) algorithms with both local and web-based MEME (Machanick and Bailey, 2011) suite.

#### Integrative ChIP-Seq and RNA-Seq analysis

HCC1359 expressing either sgCtrl or sgCIC was performed using paired-end RNA-Seq. The processed fastq files were mapped to hg19 reference genome using STAR (version 2.4) algorithm and transcript expressions were quantified using RSEM (version 1.2.29) algorithm with the default parameters. EdgeR and limma packages in R were used for differential gene expression analysis between control and CIC deficiency group, The criteria of absolute fold change >1.5 and FDR <0.2 was used to define differentially expressed genes. The promoter regions (−2000–200bp) of annotated genes from CIC binding peaks (see method “ChIP-Seq analysis”) were searched with consensus CIC octamer [T(G/C)AAT(G/A)AA] or (GGAA)n DNA motif with in-house program script, corresponding genes were intersected with DEGs, resulting in putative CIC-regulated genes.

#### Pathway analysis

Curated genes lists of CIC ChIP-Seq peaks associated with either T(G/C)AAT(G/A)AA (N = 1,185), GGAAGGAA (N = 2053), or both T(G/C)AAT(G/A)AA and GGAAGGAA (N = 157) were imported into DAVID pathway analysis (https://david.ncifcrf.gov). KEGG pathways were selected for final analysis.

#### Viability assays

Crystal violet assays were performed 10 days following drug treatments with either DMSO, trametinib, osimertinib, or vemurafenib. CellTiter-Glo (Promega) experiments were performed as per manufacturer’s protocol. Briefly, cells were plated in a 96-well plate, treated with indicated drug or control and analyzed on a Spectramax microplate reader (Molecular Devices) after 6 days of treatment. Each assay consisted of at least three replicate wells.

#### Immunostaining (IHC) with tissue microarray (TMA)

Lung adenocarcinoma tissue microarrays (TMAs) containing 100 cores of lung adenocarcinoma (LC10014a) were obtained from US Biomax and stained with CIC (Thermo Fisher Scientific) and YAP (Cell Signaling) antibodies. Each sample stained for CIC or YAP was scored as Low expression (0 or 1) or High expression (2 or 3) according to staining intensities. To generate correlation plots between CIC and YAP in the TMAs we compared the IHC scores of the paired samples using the 0–3 scoring system. TMAs were scanned and viewed using ZEISS ZEN 3.6 Imaging Software. Kendall’s rank correlation tau was used to evaluate correlation between CIC and YAP expression and a linear best-fit line was plotted using an in-house R script.

### QUANTIFICATION AND STATISTICAL ANALYSIS

Experimental data are presented as mean +/− Standard Deviation (SD). p-values derived for all in-vitro experiments and *in vivo* experiments were calculated using two-tailed student’s t-test or one-way ANOVA. CIC and YAP expression correlation was calculated using the Kendall rank correlation.

## Supplementary Material

1

## Figures and Tables

**KEY RESOURCES TABLE:** 

REAGENT or RESOURCE	SOURCE	IDENTIFIER

Antibodies		

CIC	Thermo fisher scientific	PA1-46018; RRID: AB_2291736
YAP	Cell signaling technology	Cat# 14074; RRID: AB_2650491
LATS1	Cell signaling technology	Cat# 3477; RRID: AB_2133513
LATS2	Cell signaling technology	Cat# 5888; RRID: AB_10835233
Myc-tag	Cell signaling technology	Cat# 2276; RRID: AB_331783
p44/42 MAPK (Erk1/2)	Cell signaling technology	Cat# 4695; RRID: AB_390779
Phospho-p44/42 MAPK (Erk1/2) (Thr202/Tyr204)	Cell signaling technology	Cat# 4370; RRID: AB_2315112
HSP90	Cell signaling technology	Cat# 4874; RRID: AB_2121214
Normal Rabbit IgG	Cell signaling technology	Cat# 2729; RRID: AB_1031062
ETV4 (PEA3)	Santa Cruz Biotechnology	Cat# sc-166629; RRID: AB_2278090

Bacterial and virus strains		

Stbl3™ Chemically Competent E. coli	Thermo fisher scientific	Cat# C737303

Biological samples		

Lung adenocarcinoma tissue array	US Biomax	Cat# LC10014a

Chemicals, peptides, and recombinant proteins		

Trametinib	MedChemExpress	Cat# HY-10999
Osimertinib	MedChemExpress	Cat# HY-15772
Vemurafenib	MedChemExpress	Cat# HY-12057
Verteporfin	MedChemExpress	Cat# HY-B0146
Recombinant Human EGF	Peprotech	Cat# AF-100-15
FuGENE6 transfection reagent	Promega	Cat# E2692
Lipofectamine RNAiMax transfection reagent	Thermo fisher scientific	Cat# 13778150
Blasticidin S HCl	Thermo fisher scientific	Cat# A1113903
Puromycin Dihydrochloride	Thermo fisher scientific	Cat# A1113803
T4 DNA ligase	New England Biolabs	Cat# M0202S
Phusion high-fidelity DNA polymerase	New England Biolabs	Cat# 0530L
XhoI	New England Biolabs	Cat# R0146S
KpnI-HF	New England Biolabs	Cat# R3142S

Critical commercial assays		

ECL prime western blotting system	Amersham	Cat# RPN2232
RNeasy mini kit	Qiagen	Cat# 74106
DNeasy blood & tissue kit	Qiagen	Cat# 69504
QIAGEN plasmid plus maxi kit	Qiagen	Cat# 12963
QIAquick PCR purification kit	Qiagen	Cat# 28104
Q5 Site-directed mutagenesis kit	New England Biolabs	Cat# E0554
Applied biosystems Taqman ast advanced master mix	Thermo fisher scientific	Cat# 4444557
SimpleChIP enzymatic chromatin IP kit (Magnetic beads)	Cell signaling technology	Cat# 9003
Dual-luciferase reporter assay system	Promega	Cat# E1910
CellTiter-Glo luminescent cell	Promega	Cat# G7572

Deposited data		

ChIP-seq data	Yang et al., 2017	GSE95012

Experimental models: Cell lines		

Human embryonic kidney HEK293T	ATCC	CRL-3216; RRID: CVCL_0063
Human lung adenocarcinoma H1975	ATCC	CRL-5908; RRID: CVCL_1511
Human lung adenocarcinoma H1975_10.1	Walter et al. (2013)	N/A
Human lung giant cell carcinoma HCC1359	ATCC	N/A; RRID: CVCL_5128
Human lung adenocarcinoma HCC364	ATCC	N/A; RRID: CVCL_5134
Human lung adenocarcinoma A549	ATCC	CCL-185; RRID: CVCL_0023
Human lung adenocarcinoma SW1573	ATCC	CRL-2170; RRID: CVCL_1720
Human malignant melanoma A375	ATCC	CRL-1619; RRID: CVCL_0132
Human colon adenocarcinoma HT-29	ATCC	HTB-38; RRID: CVCL_0320
Patient-derived CIC-DUX4 sarcoma NCC_CDS1_X1_C1	Oyama et al. (2017)	N/A

Experimental models: Organisms/strains		

NU/J athymic female nude mice	Jackson Laboratory	Strain #:002019; RRID: IMSR_JAX:002019

Oligonucleotides		

Oligonucleotides for RT-qPCR, ChIP-PCR and luciferase promoter assay, see supplemental tables provided	This study	This study

Recombinant DNA		

CIC (Myc-DDK-tagged) human capicua homolog	Origene	Cat# 215209
Lenti-ORF clone of CIC (mGFP-tagged) human capicua homolog	Origene	Cat# 215209L2
8xGTIIC-luciferase	Addgene	Cat# 34615
PXPR007 sgCIC-1	Addgene	Cat# 74953
PXPR007 sgCIC-2	Addgene	Cat# 74959
LV05 vector (U6-gRNA:EF1a-Cas9+FLAG-2A-Puro) containing sgLATS2	Sigma-aldrich	N/A
YAP1 shRNA #2	A gift from T. Bivona	Lin et al., 2015
YAP1 shRNA #3	A gift from T. Bivona	Lin et al., 2015

Software and algorithms		

GraphPad Prism 9.0 software	GraphPad Software	https://www.graphpad.com/scientific-software/prism/
Image J	National Institutes of Health	https://imagej.nih.gov/ij/download.html
MEME-ChIP (DREME, STREME, AME)	Machanick and Bailey (2011)	https://meme-suite.org/meme/doc/meme-chip.html?man_type=web
ZEISS ZEN 3.6 Imaging Software	Zeiss	https://www.zeiss.com/microscopy/en/products/software/zeiss-zen.html
ABI Prism 7900HT Sequence Detection System	Applied Biosystems	https://www.bu.edu/picf/files/2017/06/ABI7900HT_usersguide.pdf
Model-based Analysis of ChIP-Seq (MACS2) algorithm (version 2.2.1)	N/A	PMID:18798982
R package ChIPseeker algorithm	N/A	PMID:25765347
DAVID pathway analysis	N/A	https://david.ncifcrf.gov

## Highlights

- Capicua (CIC) suppresses the Hippo effector, *YAP1*, in human cells
- CIC regulates *YAP1* through non-consensus GGAA DNA repeat sites in human cells
- CIC and YAP cooperate to control drug resistance and tumor progression
- YAP1 inhibition overcomes CIC deficiency in human cancer
