# Supplementary material for: Capicua suppresses YAP1 to limit tumorigenesis and maintain drug sensitivity in human cancer
Source: Cell Rep. Author manuscript; Available in PMC 2022 Nov 18. (PMC9674208; doi:10.1016/j.celrep.2022.111443)
Supplement: 1 [file NIHMS1840777-supplement-1.pdf]

Cell Reports, Volume *41*

Supplemental information

Capicua suppresses *YAP1* to limit tumorigenesis and maintain drug sensitivity in human cancer

Ji Won Kim, Cuyler Luck, Wei Wu, Rovingaile Kriska Ponce, Yone Kawe Lin, Nehal Gupta, and Ross A. Okimoto

Supplementary data:

Capicua suppresses YAP1 to limit tumorigenesis and maintain drug sensitivity in human cancer.

Authors: Ji Won Kim, Cuyler Luck, Wei Wu, Rovingaile Kriska Ponce, Yone Kawe Lin, Nehal Gupta, and Ross A. Okimoto

The supplemental data includes 4 figures with legends, 2 tables, and 1 supplemental table.

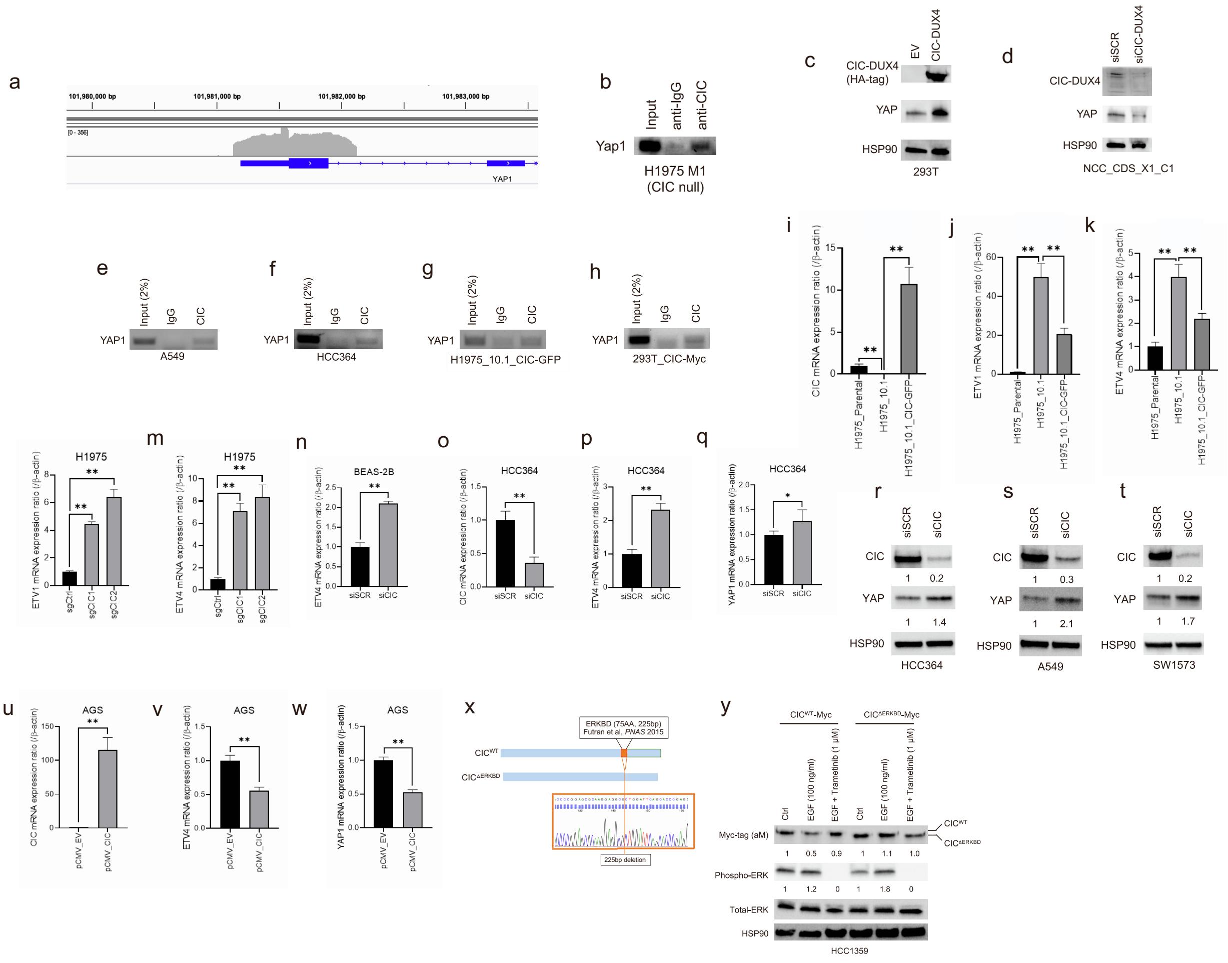

Supplementary Figure 1. Capicua containing fusion oncoprotein, CIC-DUX4, binds and regulates YAP1 expression, related to Figure 1.

(a) CIC-DUX4 peak at the proximal YAP1 regulatory element. (b) ChIP-PCR showing CIC-DUX4 occupancy of the YAP1 regulatory element in H1975_10.1 (CICWT null) cells. (c) Immunoblot of CIC-DUX4 and YAP expression in 293T cells. (d) Immunoblot of CIC-DUX4 and YAP expression in NCC_CDS_X1_C1 (endogenous CIC-DUX4) cells. (e-f) Endogenous CIC ChIP-PCR demonstrating YAP1 occupancy in A549 or HCC364 cells. (g-h) Exogenous CIC ChIP-PCR demonstrating YAP1 occupancy in H1975_10.1 expressing GFP-tagged CIC (H1975_10.1_CIC-GFP) or myc-tagged CIC overexpressing HEK293T cells. (i-k) Relative CIC (i), ETV1 (j), and ETV4 (k) mRNA expression in H1975 parental, H1975_10.1, and H1975_10.1_CIC-GFP. **p<0.01. Error bars represent S.D. (l-m) Relative ETV1 (l) and ETV4 (m) mRNA expression in H1975 cells expressing sgCtrl, sgCIC1, or sgCIC2. **p<0.01. Error bars represent S.D. (n) Relative ETV4 mRNA expression in BEAS-2B cells expressing siScramble (siSCR) control or siCIC. **p<0.01. Error bars represent S.D. (o-q) Relative CIC (o), ETV4 (p), and YAP1 (q) mRNA expression in HCC364 cells expressing siScramble (siSCR) control or siCIC. *P<0.05, **p<0.01. Error bars represent S.D. (r-t) Immunoblot of CIC and YAP in HCC364 (r), A549 (s), or SW1573 (t) cells expressing either siScramble (siSCR) control or siCIC. (u-w) Relative CIC (u), ETV4 (v), and YAP1 (w) mRNA expression in AGS (loss-of-function CIC mutation) cells expressing EV or

CICWT pCMV expression plasmids. **$p<0.01$. Error bars represent S.D. (x) Schematic diagram of the CICWT structure compared to CICΔERKBD with sanger sequencing of the in frame deletion breakpoint. (y) Immunoblot demonstrating sustained expression of CICΔERKBD compared to CICWT upon stimulation with recombinant EGF (100 ng/ml) or co-treatment with EGF and trametinib (1 μM).

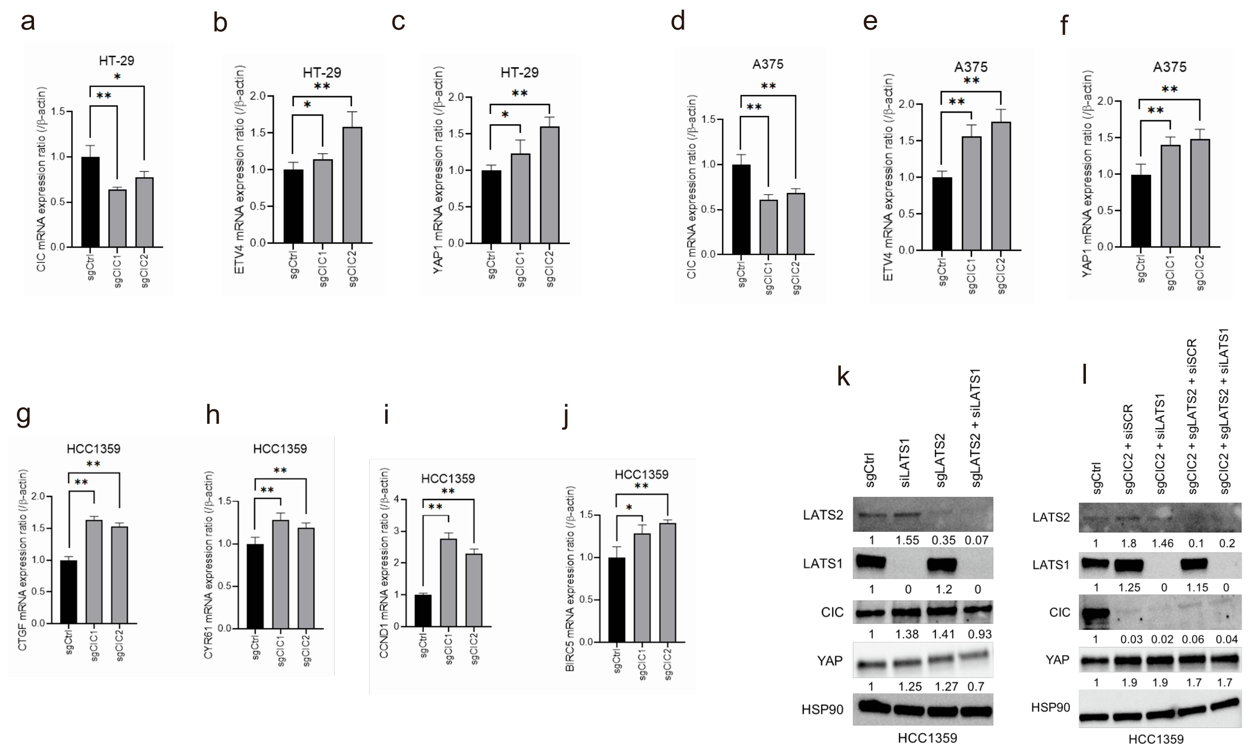

Supplementary Figure 2. CIC regulates expression of YAP1 and YAP-mediated target gene expression in a Hippo pathway independent manner, related to Figure 1.

(a-c) Relative CIC (a), ETV4 (b), and YAP1 (c) mRNA expression in HT-29 colorectal adenocarcinoma cells expressing sgCtrl, sgCIC1, or sgCIC2. *$p<0.05$, **$p<0.01$. Error bars represent S.D. (d-f) Relative CIC (d), ETV4 (e), and YAP1 (f) mRNA expression in A375 melanoma cells expressing sgCtrl, sgCIC1, or sgCIC2. **$p<0.01$. Error bars represent S.D. (g-j) Relative mRNA expression of CTGF (g), CYR61 (h), CCND1 (i), and BIRC5 (j), target genes of YAP, in HCC1359 cells expressing sgCtrl, sgCIC1, or sgCIC2. *$p<0.05$, **$p<0.01$. Error bars represent S.D. (k-l) Immunoblots of LATS2, LATS1, CIC, and YAP in HCC1359 cells (k) or HCC1359 cells stably expressing sgCIC2 (l) with either sgLATS2, siLATS1, or both sgLATS2 and siLATS1.

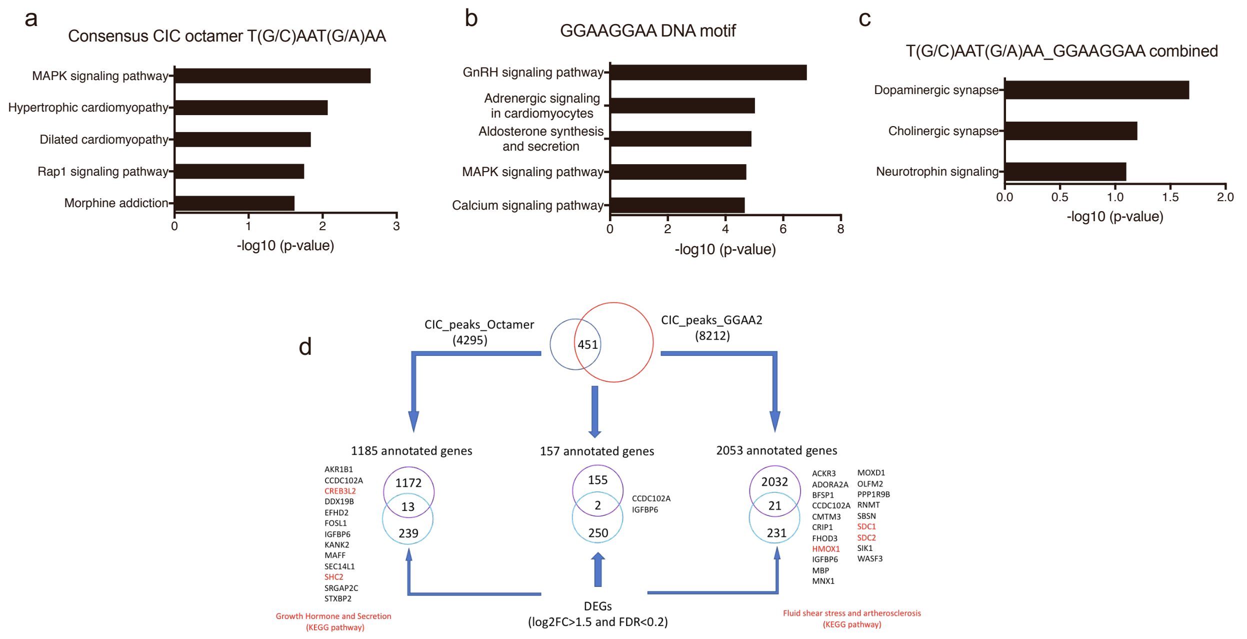

Supplementary Figure 3. CIC regulates diverse cellular functions through consensus and non-consensus DNA-binding sites, related to Figure 2.

(a-c) KEGG Pathway analysis using annotated CIC ChIP-Seq genes associated with the consensus T(G/A)AAT(G/A)AA (a), non-concensus (GGAAx2+) (b), or both T(G/A)AAT(G/A)AA and GGAAx2+ (c) motifs. (d) Integration of ChIP-Seq analysis with RNA-Seq data from HCC1359 cells +/- CIC expression.

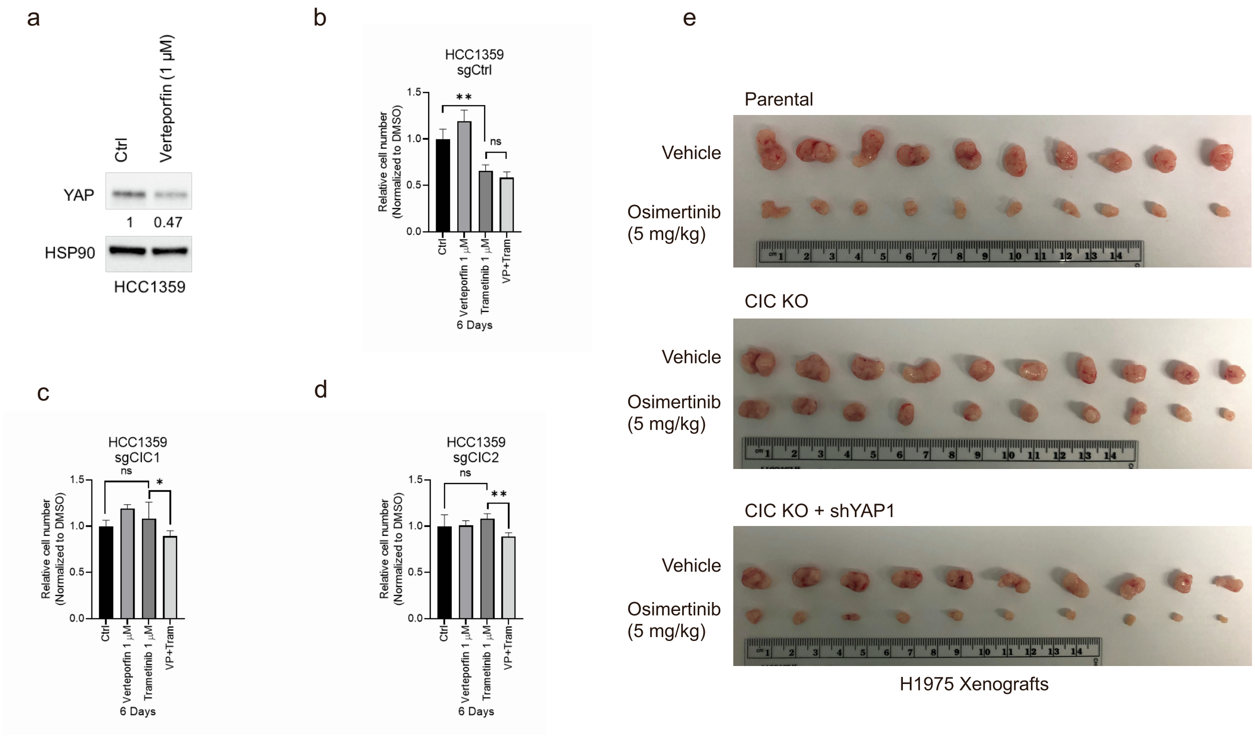

Supplementary Figure 4. YAP inhibition restores sensitivity to MAPK-targeting agents, related to Figures 3 and 4.

(a) Immunoblots of YAP and HSP90 in HCC1359 cells treated with DMSO or verteporfin (1 μM) for 24 hours. Representative of two independent experiments. (b-d) Relative cell number of HCC1359 cells expressing either sgCtrl (b), sgCIC1 (c), or sgCIC2 (d) and treated for 6 days with verteporfin, trametinib, or combination verteporfin + trametinib. *p<0.05, **p<0.01. Error bars represent S.D. (e) Explanted tumor xenografts from mice harboring H1975 parental, H1975 CIC KO, or H1975 CIC KO + shYAP1 +/- osimertinib treatment.

Supplementary Table 1. Consensus and Non-Consensus DNA binding motifs mapped to CIC ChIP-Seq peaks, related to Figure 2.

DNA binding sequence	# of genomic locations	% of all CIC peaks	# of annotated genes
GGAA	77911	98 %	14308
GGAAGGAA	8212	10 %	2053
GGAAGGAAGGAA	190	0.24 %	45
TGAATGGA	1505	1.89 %	400
TCAATGAA	645	0.81 %	174
TCAATGGA	697	0.87 %	209
TGAATGAA	1620	2.0 %	402
T(G/C)AAT(G/A)AA	4295 (no duplicates)	5.63 %	1185

Supplementary Table 2. List of genes contain both GGAAx2+ and T(G/C)AAT(G/C)AA CIC binding motifs, related to Figure 2.

Gene symbol						
SNORD115-2	SOGA1	PDLIM3	AFF3	CRTAC1	SUMO2	SLX4
SNORD115-9	IGSF23	APOBEC3C	LAIR1	LGI2	SOX12	SORBS2
SNORD115-10	PTPRVP	BIN1	MGMT	RNLS	SRD5A1	GPT2
SNORD115-11	JAKMIP1	CECR2	DDX12P	BCAS4	TCF15	MGARP
SNORD115-17	DCTD	ZDHHC22	SNX29P2	PRKAR1B	TEAD4	ZNF577
LIN28B-AS1	DCTN1	KLK9	MUC2	INAVA	TLE3	MIR503HG
ZBTB42	DNMT3B	SLC25A42	ZFHX3	MAPK13	TNS1	KIAA1671
LOC100128993	KRT24	GRB10	NEUROD1	PRMT8	TP73	STX10
CDH4	ERG	LGALSL	NPY5R	KLK10	CCDC175	TNFRSF25
LINC00629	FAT1	NT5C	NTSR1	PSMD1	NR1H2	APLN
CDH8	CYB561A3	HMGB3	MIR424	PTEN	CACNA1A	CCND3
POP7	RASGEF1A	NR4A1	OCRL	MIR503	ARMC7	PAPSS2
DNAJA2	SHANK2	SNORD109B	P4HB	MICAL3	CPSF7	CCDC102A
BAIAP2	MTUS2	OTOG	JPT1	PTPN7	B9D2	TGFBRAP1
ADARB2	SLC7A8	ECEL1P2	TMEM216	PTPRE	EEPD1	OPN4
CEBPE	LILRA4	IGFBP6	PCSK2	PVR	DUSP16	NAPSA
BVES	ALPP	TNFRSF9	SCARA3	SH2D4A	CAMK2A	NCOR2
NACC1	NAPSB	IPW	RAB6B	TMEM91	CAMK2G	PIEZO1
LMTK3	C2orf72	CERKL	NECAB2	LIPK	ABHD17A	ARHGEF11
CCR7	NGEF	KCNJ6	GNB1L	ANHX	DYSF	
ANKS3	C20orf194	CCDC9B	FBLIM1	CERK	TRAPPC9	
C1orf216	CYTH4	SBK1	PAQR5	SLC4A1	TKTL2	
CRABP2	BRPF3	LIN28B	NSUN2	SLC7A1	SOAT2	

Supplementary Table 3. List of oligonucleotides, related to STAR Methods

Gene name	Species	Primer sequences
YAP1 ChIP-PCR	Human	5’-GGACTCGGAGACCGACCTGGAG-3’
		5’-GCTCCGGCGGCTTGAAGAAGG-3’
CIC$^{\Delta ERKBD}$ mutant	Human	5’-CTGGATTCAGCACCCGAGGACC-3’
		5’-CGCCTCCTTGCGCTCCGG-3’
pGL4.10-YAP1 (-1533/+86)	Human	5’-GATCGGTACCAGTCATGGGTGTC-3’
		5’-GATCCTCGAGAGCTCGTTGCCTTTC-3’
pGL4.10-YAP1 (-461/+86)	Human	5’-TATCAAGATCTGGCCTCG-3’
		5’-GCCGAAAGAAGTGGAGAG-3’
pGL4.10-YAP1 (-305/+86)	Human	5’-TATCAAGATCTGGCCTCG-3’
		5’-CAAACGATGGGTCCAATC-3’
pGL4.10-YAP1 (-4/+86)	Human	5’-TATCAAGATCTGGCCTCG-3’
		5’-CCCGACTGAGACAGAAAC-3’
pGL4.10-YAP1 (-256/-5)	Human	5’-CGCAGCCGCCGCCAGGGAAAAG-3’
		5’-CAGCCGGGCAGGGGCCCG-3’
CIC siRNA	Human	Cat# L-015185-01-0005 (GE Dharmacon)
LATS1 siRNA	Human	Cat# L-004632-00-0005 (GE Dharmacon)
CIC Taqman gene expression assays	Human	Hs00943425_g1
YAP1 Taqman gene expression assays	Human	Hs00902708_g1
ETV1 Taqman gene expression assays	Human	Hs00951951_m1
ETV4 Taqman gene expression assays	Human	Hs00383361_g1
CTGF Taqman gene expression assays	Human	Hs00170014_m1
CYR61 Taqman gene expression assays	Human	Hs00155479_m1
CCND1 Taqman gene expression assays	Human	Hs00765553_m1
BIRC5 Taqman gene expression assays	Human	Hs04194392_s1
β-actin Taqman gene expression assays	Human	Hs01060665_g1
